# Visual Influence on Auditory Perception of Vowels by French-Speaking Children and Adults

**DOI:** 10.3389/fpsyg.2022.740271

**Published:** 2022-02-25

**Authors:** Paméla Trudeau-Fisette, Laureline Arnaud, Lucie Ménard

**Affiliations:** ^1^Laboratoire de Phonétique, Université du Québec à Montréal, Montreal, QC, Canada; ^2^Centre for Research on Brain, Language and Music, Montreal, QC, Canada; ^3^Integrated Program in Neuroscience, McGill University, Montreal, QC, Canada

**Keywords:** speech perception, adults, children, sensorimotor maturation, audiovisual interaction

## Abstract

Audiovisual interaction in speech perception is well defined in adults. Despite the large body of evidence suggesting that children are also sensitive to visual input, very few empirical studies have been conducted. To further investigate whether visual inputs influence auditory perception of phonemes in preschoolers in the same way as in adults, we conducted an audiovisual identification test. The auditory stimuli (/e/-/ø/ continuum) were presented either in an auditory condition only or simultaneously with a visual presentation of the articulation of the vowel /e/ or /ø/. The results suggest that, although all participants experienced visual influence on auditory perception, substantial individual differences exist in the 5- to 6-year-old group. While additional work is required to confirm this hypothesis, we suggest that auditory and visual systems are developing at that age and that multisensory phonological categorization of the rounding contrast took place only in children whose sensory systems and sensorimotor representations were mature.

## Introduction

In neurotypical individuals, face-to-face communication is multisensory (Rosenblum, 2008a,b). In addition to body movements and facial expressions, effective communication relies heavily on multisensory information, such as auditory, visual, and proprioceptive cues (Stein and Stanford, 2008; Stein et al., 2014). Multisensory processing is crucial for efficient perception, as it optimizes brain functions and reduces perceptual ambiguity (Stein et al., 2014; Gori, 2015).

From a developmental perspective, several studies provide evidence that children do not display adult-like multisensory processing until late childhood (Gori et al., 2008; Barutchu et al., 2009; Burr and Gori, 2012). First, since sensory systems are not mature at birth, but evolve and are calibrated throughout childhood (Burr and Gori, 2012), multisensory processing constantly adapts to different kinds of inputs (Birch and Lefford, 1963; Yu et al., 2010). Second, the brain areas shown to be involved in multisensory processing are not operational at birth but develop with experience (Stein et al., 2014). Despite its crucial role in speech perception and its continuing refinement in the first years of life, very little is known about the development of multisensory processing in the specific area of speech.

This study is part of a larger project investigating the development of such perceptual processes in school-aged children. In a recent paper (Trudeau-Fisette et al., 2019), we investigated a specific case of multisensory processing, namely, the interaction between auditory and somatosensory input during vowel perception in children and adults. More specifically, 10 synthesized vowels equally stepped on a continuum from /e/ (as in “fée” *fairy*) to /ø/ (as in “feu” *fire*) were presented in the auditory modality to francophone adults and children ranging in age from 4 to 6 years old. The participants’ task was to categorize the sounds they perceived as either /e/ or /ø/. In some trials, a facial skin stretch applied by a mechanical device on the participant’s cheeks was synchronized with the audio signal, mimicking the articulation normally associated with the production of the vowel /e/. The data showed that the effects of somatosensory feedback on auditory vowel categorization were reduced in children compared to adults. Our results thus suggest that preschool-aged children do not combine auditory and somatosensory information in the same way as adults do. In the current follow-up paper, we focus on the interaction between the auditory and visual sensory modalities in the perception of the same vowel continuum in francophone children and adults.

### The Development of Audiovisual Interaction in Speech Perception

Substantial work has shown evidence of early sensitivity to auditory and visual interaction in speech perception. For instance, it has been shown that babies show facial mimicking skills after only a few days of life (Meltzoff and Moore, 1983) and that they are able, after a couple of months, to recognize whether or not information they receive through auditory and visual channels is compatible (Dodd, 1979; Kuhl and Meltzoff, 1982, 1984; Legerstee, 1990; Patterson and Werker, 1999; Burnham and Dodd, 2004). Prelinguistic infants are also sensitive to the famous McGurk effect, whereby an auditory stimulus /ba/ dubbed on a visual stimulus/ga/triggers the perception of/da/ (McGurk and MacDonald, 1976). However, in their original work, McGurk and MacDonald (1976) observed that anglophone children (aged 3–4 and 7–8) were generally less subject to audiovisual illusions than adults (see also Massaro et al., 1986). Dupont et al. (2005) also reported a reduced influence of visual input on audiovisual consonant perception in French-speaking children aged 4 and 5 years compared to adults. In incongruent stimuli (were the visual signal corresponded to a different phoneme than the auditory signal), children relied more frequently on the auditory signal only than adults did. Several papers have since confirmed that, during the first decade of life, children do not integrate auditory and visual cues as much as adults do (Hockley and Polka, 1994; Desjardins and Werker, 2004; Tremblay et al., 2007; Burr and Gori, 2012; Knowland et al., 2014).

Another common manifestation of the interaction between auditory and visual cues in speech perception is multisensory enhancement, whereby identification scores are greater in the audiovisual condition than in either the auditory or the visual condition (see Stein et al., 2014 for a discussion of this process in MSI). This audiovisual enhancement (or audiovisual gain) is found in quiet conditions (Gagne et al., 1994; Robert-Ribes et al., 1998) as well as in contexts where the auditory signal is degraded (Grant and Seitz, 2000; Dohen and Loevenbruck, 2009). Indeed, in noisy environments, visual information on the speech articulators helps shape the overall perception of speech signals by recovering part of the information that is lost from the auditory channel. Similarly to the pattern found for the McGurk effect, Ross et al. (2011) found that children (aged 5–14 years old) had a smaller gain in correct identification scores than adults when exposed to audiovisual signals compared to auditory signals in low signal-to-noise ratios (SNRs; see also Barutchu et al., 2010). Along the same lines, Wightman et al. (2006) showed that, until 9 years of age, visual information is not used to recover masked auditory signals.

To summarize, past findings indicate that, while prelinguistic infants are able to distinguish bimodal from unimodal speech stimuli and show a preference for compatible information, children do not attach as much weight to visual sensory cues as adults do in incongruent audiovisual conditions or degraded auditory conditions. While the literature indicates that MSI processes in general require sensory experiences and brain maturation (Gori et al., 2008; Hillock et al., 2011; Stein et al., 2014), it is suggested that some form of audiovisual interaction exists early in life.

### The Case of the Rounding Contrast

Perceptual facilitation of audiovisual information is largely due to the fact that information recovered by the ear and eye is complementary: while auditory cues mainly convey voicing and manner, visual inputs transmit information about place of articulation and rounding (Macleod and Summerfield, 1987; Robert-Ribes et al., 1998; Ménard, 2015; Peelle and Sommers, 2015). In a study of vowel perception in French in various sensory conditions (auditory alone, visual alone, and audiovisual) at different noise levels, Robert-Ribes et al. (1998) proposed robustness scales for vowel features (the higher the correct identification score in noisy conditions, the greater the robustness). In the audio channel, height is the most robust feature, followed by place of articulation, which in turn is more robust than rounding. In the visual channel, rounding is the most robust feature, followed by height, while place of articulation is the least robust feature.

Because of its visual saliency, the contrast between rounded vowels and unrounded vowels is ideal for studying audiovisual interactions in speech perception, particularly in languages like French, Dutch, or Swedish, in which this feature is phonologically relevant. In French, Lisker and Rossi (1992) instructed experts to rate vowel rounding based on an auditory presentation of vowels, sometimes accompanied by unmatched visually articulated vowels. Despite clear acoustic signals denoting rounded vowels, most listeners were influenced by the visually presented unrounded vowels (although the extent of the interaction varied across participants). Furthermore, Traunmüller and Öhrström (2007) presented nonsense syllables containing unrounded vowels (/gig/ and /geg/) and rounded vowels (/gyg/ and /gøg/) to adult Swedish participants. Their task was to identify the syllable they perceived. In some trials, the auditory and visual parts of the stimuli corresponded to similar rounding values (congruent stimuli) while in others, the two modalities denoted different values (incongruent stimuli). Responses to the incongruent stimuli suggested that visually indexed rounding is heavily weighted in listeners’ perception: a stimulus in which the auditory part is unrounded and the visual part is rounded is generally perceived as rounded. In a later paper, Valkenier et al. (2012) combined visually articulated Dutch vowels contrasting in terms of height and rounding to congruent and incongruent auditory signals of the vowels, mixed with noise. Native Dutch adult listeners were instructed to identify the vowel they perceived. The results showed an audiovisual facilitation effect, as correct identification was enhanced by congruent audiovisual presentation. On the contrary, incongruent presentation degraded correct identification.

Although the influence of visual cues on the auditory perception of the rounding feature in languages like French has been established, nothing is known about its development in children. In this paper, we report on an experiment carried out to investigate whether processing of auditory and visual information occurs in preschool-aged children in the same way as it does in adults. As in our previous study of auditory and somatosensory interaction (Trudeau-Fisette et al., 2019), we focused on the perception of the unrounded/rounded vowel pair /e/-/ø/ in Canadian French. Based on the previous work on the developmental time course of MSI processes presented in Section “The Development of Audiovisual Interaction in Speech Perception”, it is expected that the effect of vision on the perception of rounding contrasts will be reduced in school-aged children compared to adults.

## Materials and Methods

### Participants

Thirty young adults and 30 children were recruited. After excluding three adults and seven children due to equipment malfunction (two adults and two children), non-completion (three children), or inability to perform the task (one adult and two children), we were left with 27 adults (aged 19–30; mean = 26.3, 13 females) and 23 children (aged 5–6; mean = 5.6, 15 females). At that age, the phonemic categories under study (/e/ and /ø/) are well mastered (Ménard et al., 2007).

All participants were native speakers of Canadian French and were tested for pure-tone detection threshold using an adaptive method (DT < 25 dB HL at 250, 500, 1,000, 2,000, 4,000, and 8,000 Hz). Every participant (or their parents) reported that they had never had speech, language, neurological, or psychological disorders. They also reported having normal or corrected-to-normal vision. Every participant (or their parents) gave written informed consent to participate in the experiment. The research protocol was approved by Université du Québec à Montréal’s Institutional Review Board (no. 2015-05-4.2).

### Experimental Procedure

The experiment consisted of an audiovisual identification test. The auditory stimuli corresponded to 10 equally stepped synthesized five-formant vowels on the /e–ø/ continuum. The vowels were synthesized using the Maeda model (Maeda, 1989). First, prototypical formant and bandwidth values for French /e/ and /ø/ were determined for the model (Ménard et al., 2004). For each of the vowels on the continuum, the values of the first and fifth formants were fixed (F1 = 364 Hz and F5 = 4,000 Hz), while the second, third, and fourth formant values (in Hertz) were interpolated from the values of the two endpoints /e/ and /ø/ (see Table 1). Formant bandwidth values were as follows: B1 = 48 Hz, B2 = 55 Hz, B3 = 60 Hz, B4 = 50 Hz, and B5 = 100 Hz. Each stimulus lasted 600 milliseconds and had a mean fundamental frequency of 130 Hz.

**Table 1:** Values of the second, third, and fourth formants (in Hz) of the synthesized stimuli used in the perceptual task.

Stimulus number	F2	F3	F4
1	1,922	2,509	3,550
2	1,892	2,469	3,500
3	1,862	2,429	3,450
4	1,832	2,389	3,400
5	1,802	2,349	3,350
6	1,772	2,309	3,300
7	1,742	2,269	3,250
8	1,712	2,229	3,200
9	1,682	2,189	3,150
10	1,652	2,149	3,100

*For all stimuli, F1 = 364 Hz, F5 = 4,000 Hz, B1 = 48 Hz, B2 = 55 Hz, B3 = 60 Hz, B4 = 50 Hz, and B5 = 100 Hz. Stimulus 1 is the prototypical /e/ and stimulus 10 is the prototypical /ø/*.

**Figure 1:**
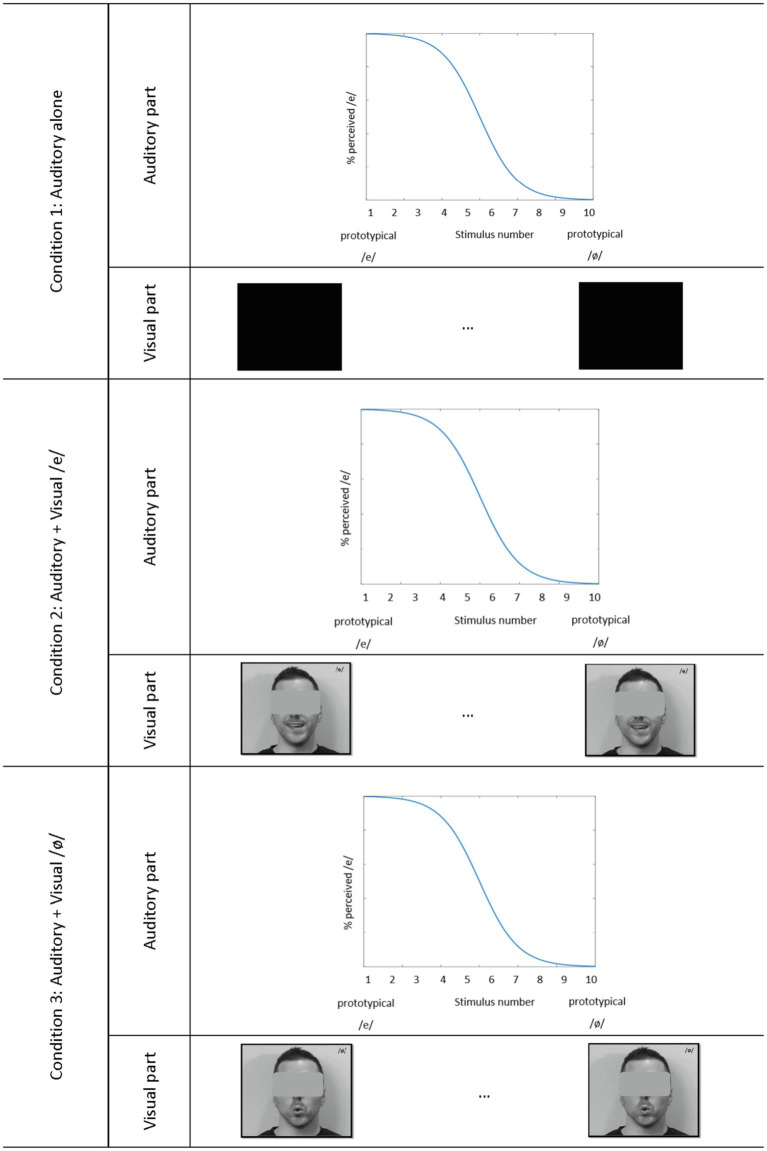
Schematic representation of the audiovisual stimuli in the three conditions.

In our experiment, the identification scores in various audiovisual conditions involving different visual components mixed with the same auditory stimuli will be compared. The auditory modality will be considered the dominant modality. Following a method used previously in speech perception (Ito et al., 2009; Trudeau-Fisette et al., 2019), the synthesized vowels were presented either in an auditory-only (AO) condition or simultaneously with a visual presentation of the prototypical articulation of the vowel /e/ or /ø/ (see Figure 1 for a schematic description of the audiovisual conditions). The visual signals were obtained from an adult French Canadian male speaker producing the two vowels /e/ and /ø/. The use of prototypical visual stimuli has been shown to be efficient in evaluating audiovisual speech perception in a young population (Kuhl and Meltzoff, 1982; Patterson and Werker, 2003; Yeung and Werker, 2013). Several repetitions of the vowels were obtained with instructions to start and end with a neutral position. The best occurrence of each vowel was selected by the experimenter. In one condition (AV /e/), each of the 10 synthesized auditory stimuli of the /e/-/ø/ continuum was manually mapped into the muted visual articulation of /e/. In the other condition (AV /ø/), each auditory stimulus was combined with the visual articulation of /ø/. Participants were asked to identify the vowel they perceived and were forced to choose between /e/ and /ø/. To ensure that the children understood the difference between the two vowel choices, the vowel /e/ was represented by an image of a fairy (/e/ as in *fée*) and the vowel /ø/ was characterized by an image of a fire (/ø/ as in *feu*). Adults were asked to use the right and left arrows of the computer keyboard to indicate their responses. Children pointed to the image corresponding to their answers (placed right in front of them on the top left and right corners of the laptop) and the experimenter selected the corresponding keyboard key. A practice round was conducted with each participant to ensure that they understood the task.

The stimuli were presented in 18 blocks of 10 trials each. Within each block, all members of the 10-step continuum were presented in a randomized fashion. For one-third of the trials, only the auditory stimulus was presented (Auditory Only). For the other two-thirds of the trials, the visual articulation corresponding to the vowels /e/ and /ø/ was also presented randomly. Alternate blocks of unimodal and bimodal conditions (Audiovisual /e/ condition and Audiovisual /ø/ condition altogether) were presented to the participants. In total, 180 perceptual judgments were collected, 60 in the auditory-only condition, and 120 in the combined audiovisual conditions.

### Data Analysis

For each participant, stimulus, and condition, we calculated the percentage of /e/ responses. Although reaction time (RT) could not be analyzed due to differences in the experimental procedures between the two groups, it was used to exclude responses where RT was ±2 standard deviations from the blocks’ mean RT. In doing so, we sought to eliminate categorical judgments for which the participants were no longer in a position to properly respond to the task (less than 2.2 and 1.1% of all responses were excluded for children and adults, respectively). Excluded responses were fairly distributed across speakers and conditions; no more than 3% of the responses were discarded for each individual. While perceptual categorization of speech targets is often analyzed through psychometric functions (e.g., 50% crossover boundaries and labeling slopes), responses collected from children, for whom the categorical boundary was rarely crossed in the audiovisual conditions, prevent us from using these paradigms to describe the obtained results. Therefore, each answer given (9,000 perceptual judgments collected from 50 participants) was fitted into a linear mixed-effects model (LMEM; using the *lme4* package in R) in which the fixed factors were stimulus (the 10-step continuum), group (adult or children), and condition [Auditory Only (AO), Audiovisual /e/ (AV /e/), or Audiovisual /ø/ (AV /ø/)], and the random factor was the individual participant. *Post-hoc* analyses were performed using multiple comparisons (using the *multcomp* package in R). Values of *p* were corrected using the Bonferroni method. It should be specified that, since we were interested in the influence of vision on the auditory phonemic categorization of the rounding contrast, only individuals with a typical psychometric curve in the AO condition (those for whom the endpoint stimuli belonged to the two different phonological categories) were included in the analyses. As mentioned previously, two children were excluded because of their “inability to perform the task.”

## Results

### Mean Perceptual Scores Across Conditions and Groups

**Figure 2:**
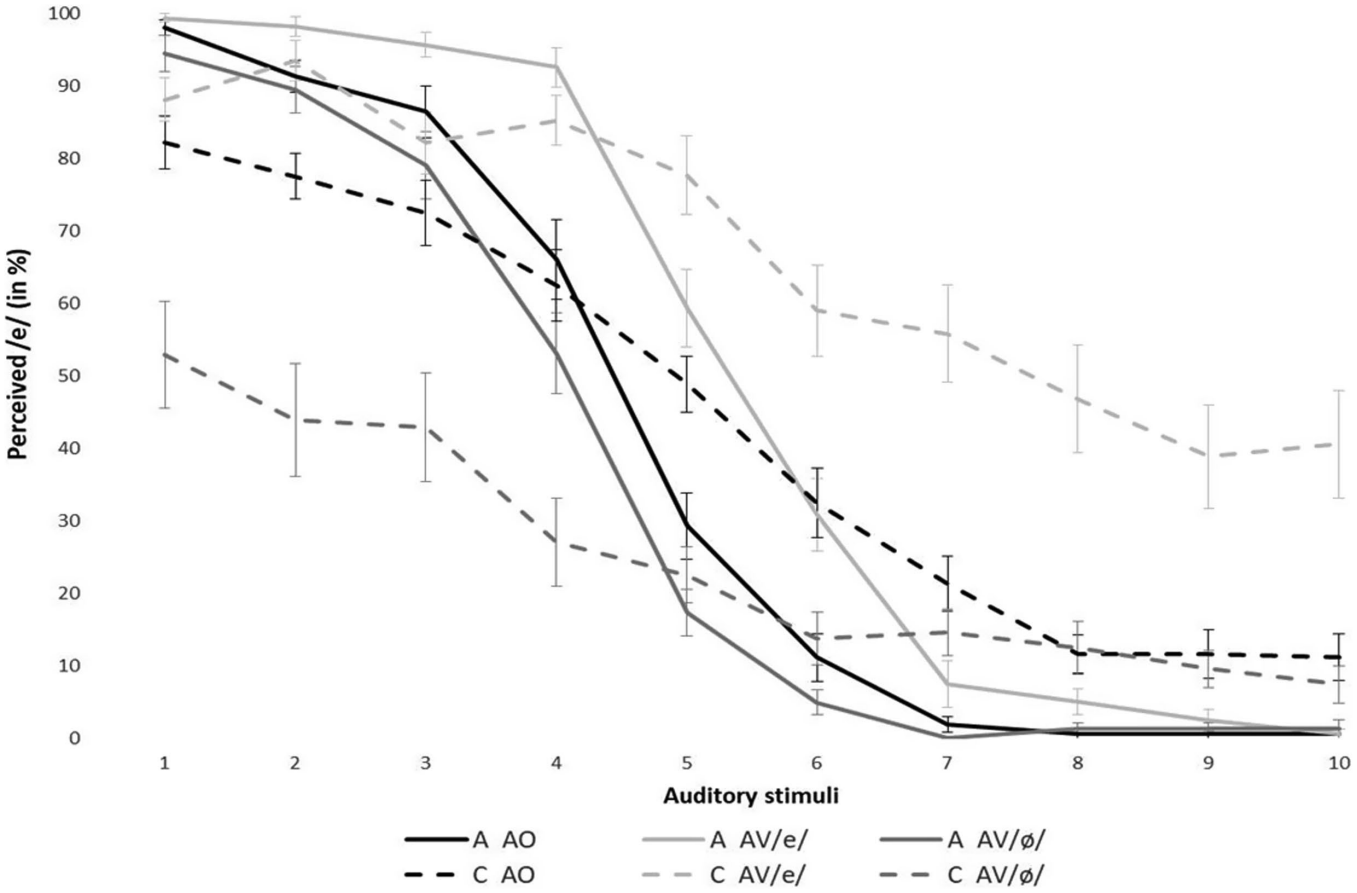
Mean percentage identification of the vowel [e] for stimuli on the [e] – [ø] continuum across speaker groups and experimental conditions. Error bars indicate standard errors (A, Adults; C, Children; AO, auditory only; and AV, audiovisual).

The mean percentage of /e/ responses for each stimulus is shown in Figure 2. Data are averaged across speakers, within each group (Adults and Children) and experimental condition (AO, AV/e/, and AV/ø/).

In addition to the expected main effect of stimulus [*χ*^2^(1) = 4,360, *p* < 0.001], the LMEM revealed significant effects of group [*χ*^2^(1) = 4.88, *p* < 0.05] and condition [*χ*^2^(2) = 772.14, *p* < 0.001]. Overall, this means that, regardless of the experimental condition, children and adults labeled the perceived stimuli differently and that, for both groups, the three experimental conditions led to different categorization scores.

Significant interactions between group and stimulus [*χ*^2^(1) = 482.92, *p* < 0.001], group and condition [*χ*^2^(2) = 197.21, *p* < 0.001], and condition and stimulus [*χ*^2^(2) = 51.73, *p* < 0.001] were also revealed. In the AO condition, children and adults had significantly different identification scores. For the prototypical stimuli, children had lower identification scores than adults for /e/-like stimuli (stimuli 1–3). Conversely, for /ø/-like stimuli (stimuli 8–10), children perceived /e/ more than adults (thus, less /ø/). The perception of several ambiguous stimuli (stimuli 5–7) also yielded significantly higher identification scores for /e/ in children than adults, suggesting a less categorical shape of the perception function. More importantly, a significant three-way interaction between group, condition, and stimulus [*χ*^2^(7) = 785.80, *p* < 0.001] was found, revealing that, for various stimuli, audiovisual presentation affected the perceptual categorization of the speech target differently for children and adults.

*Post-hoc* tests indicated that, in children, responses corresponding to the perception of stimuli 1–6 (/e/-like to ambiguous) presented with the visual articulation of the vowel /ø/ (dashed dark gray line) were associated with the label /ø/ significantly more than when perceived only auditorily (1: *z* = −0.815, *p* < 0.001; 2: *z* = −9.256, *p* < 0.001; 3: *z* = −7.242, *p* < 0.001; 4: *z* = −7.746, *p* < 0.001; 5: *z* = −5.348, *p* < 0.001; and 6: *z* = −3.875, *p* < 0.01). Likewise, categorization of stimuli 4–10 (ambiguous to /ø/-like) presented under the bimodal /e/ condition (dashed light gray line) was associated with the vowel /e/ more than in the AO condition (4: *z* = 4.932, *p* < 0.001; 5: *z* = 5.888, *p* < 0.001; 6: *z* = 5.539, *p* < 0.001; 7: *z* = 8.321, *p* < 0.001; 8: *z* = 10.225, *p* < 0.001; 9: *z* = 8.347, *p* < 0.01; and 10: *z* = 9.887, *p* < 0.01). In adults, only the visual /e/ condition (solid light gray line) on ambiguous auditory targets (stimuli 4, 5, and 6) led to a significant change in categorization (4: *z* = 6.439, *p* < 0.001; 5: *z* = 6.259, *p* < 0.001; and 6: *z* = 4.452, *p* < 0.001).

**Figure 3:**
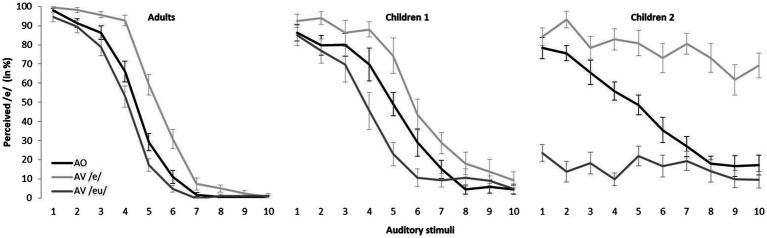
Mean percentage identification of the vowel [e] for stimuli on the [e] – [ø] continuum across speaker groups and experimental conditions. Error bars indicate standard errors.

To better evaluate the effect of visual stimuli on children’s auditory perception, we divided the young participants into two groups based on their categorization pattern in the AV conditions. Since we are interested in the perception of the rounding contrast, we divided the child participants based on the presence or absence of a categorical distinction in the AV conditions. Figure 3 displays the overall percentage of /e/ responses for each stimulus, according to the three groups: Adults, Children 1 (C1), and Children 2 (C2). C1 consisted of 11 (mean age: 5.8) children whose categorical slopes crossed the 50% boundary in both AV conditions. C2 was composed of 12 children (mean age: 5.4) for whom the 50% boundary was not crossed in at least one of the AV conditions. For 5 of those 12 individuals, the 50% boundary was not crossed in any of the AV conditions. Interestingly, the 50% boundary was crossed in the AV /e/ condition for only six of the remaining children while it was crossed in the AV /ø/ condition for only a single child. As Figure 3 shows, individuals from all groups had a typical psychometric curve in the AO condition. The data presented in Figure 3 reveal that, rather than performing at the chance level, children adopted two completely different behavioral patterns. The first group of children, C1 (middle graph in Figure 3) displayed a clear categorical distinction between /e/ and /ø/ in the three experimental conditions. However, the C2 group of children (right-hand graph in Figure 3) did not display a response pattern in the two AV conditions consistent with the categorical perception of /e/ and /ø/: unlike the children in the C1 group, those in the C2 group made responses dominated by visual input (either /e/ or /ø/, depending on the condition). To account for the differences between these groups, LMEM analyses were computed where the fixed factors were stimulus (the 10-step continuum), group (adult, C1, or C2), and condition [AO, AV /e/, or AV /ø/], and the random factor was the individual participant. While no main effect of group was detected [*χ*^2^(1) = 3.049, *p* > 0.05], significant interactions between group and stimulus [*χ*^2^(1) = 161.27, *p* < 0.001], group and condition [*χ*^2^(2) = 44.75, *p* < 0.001], and group, stimulus, and condition were identified [*χ*^2^(7) = 267.14, *p* < 0.001].

### Visual Gain on Categorization Scores

**Figure 4:**
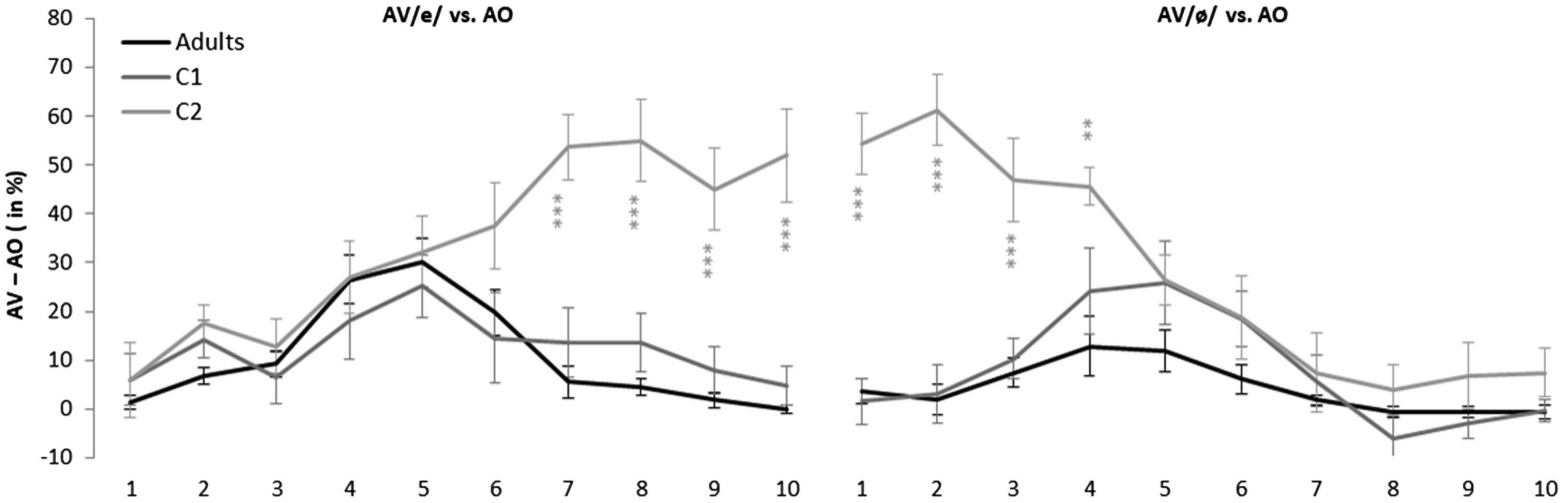
Mean visual influence on the categorization of auditory stimuli on the [e] – [ø] continuum across speaker groups and experimental conditions. Error bars indicate standard errors. ^**^p < 0.01 and ^***^p < 0.001.

In order to better investigate the weight of experimental condition in the three-way interaction displayed in Figure 3, the difference between the identification scores in the two AV conditions relative to the AO condition was computed. This difference, corresponding to the visual gain, is shown in Figure 4 for the three groups. The difference in categorization between the AV/e/ and AO conditions is shown in the left-hand panel, while the difference between the AV/ø/ and AO conditions is displayed in the right-hand panel. For the sake of clarity, significance is shown only with asterisks, but detailed *z* values for group differences are presented in Table 2.

**Table 2:** Summary of *z* values and significance levels of visual influence on the categorization of stimuli 1–10 according to audiovisual condition (cases where no significant difference is found are denoted by the symbol “–”).

Stimuli	AV/e/ vs. AO			AV/ø/ vs. AO		
	Adults vs. C1	Adults vs. C2	C1 vs. C2	Adults vs. C1	Adults vs. C2	C1 vs. C2
*/e/-like*	1	–	–	–	–	9.392, *p* < 0.001	−8.584, *p* < 0.001
	2	–	–	–	–	10.984, *p* < 0.001	−9.18, *p* < 0.001
	3	–	–	–	–	6.950, *p* < 0.001	−5.398, *p* < 0.001
*Ambiguous*	4	–	–	–	–	4.289, *p* < 0.01	–
	5	–	–	–	–	–	–
	6	–	–	–	–	–	–
	7	–	6.699, *p* < 0.001	−5.253, *p* < 0.001	–	–	–
*/ø/−like*	8	–	9.018, *p* < 0.001	−6.582, *p* < 0.001	–	–	–
	9	–	8.519, *p* < 0.001	−6.092, *p* < 0.001	–	–	–
	10	–	10.321, *p* < 0.001	−7.327, *p* < 0.001	–	–	–

Overall, Figure 4 and Table 2 show that Adults, and C1’s categorization patterns are quite similar. In fact, no differences were found between the Adult and C1 groups, regardless of AV condition and auditory stimulus. Although C2 classifications form a completely different pattern, it seems that visual inputs affect all three groups in a similar way for stimuli 1–6 (under AV /e/ condition, left-hand panel) and stimuli 5–10 (under AV /ø/ condition, right-hand panel). However, Figure 4 clearly shows that substantial group differences were found and that those differences reflect the congruity of auditory and visual information.

When ambiguous auditory stimuli were perceived, all three groups were affected by visual information. Indeed, as Figure 4 shows, large differences in categorization were observed between the AV conditions and the AO condition for stimuli 4–7. Setting aside group C2’s particular classification pattern, it can be seen that, compared to C1 (dark gray lines), for whom both AV conditions led to considerable changes, the visual cues barely affected adults’ classifications of ambiguous auditory stimuli in the AV/ø/ condition (black line, right-hand panel). As a matter of fact, unlike both groups of children, adults were influenced only by seeing the articulatory movement of the vowel /e/. This pattern is clearly illustrated in Figure 3 (left-hand panel) and Table 3, where *z* values corresponding to the differences in categorization between the two AV conditions and the AO condition are presented, this time according to the three experimental groups. The effect of visual information on the categorization of ambiguous stimuli is highlighted in light gray.

**Table 3:** Summary of *z* values and significance levels of visual influence on the categorization of stimuli 1–10.

Stimuli		Adults		Children 1 (C1)		Children 2 (C2)		AV/e/ vs. AO	AV/ø/ vs. AO	AV/e/ vs. AO	AV/ø/ vs. AO	AV/e/ vs. AO	AV/ø/ vs. AO
*/e/−like*	1	–	–	–	–	–	−11.584, *p* < 0.001
	2	–	–	–	–	–	−12.932, *p* < 0.001
	3	–	–	–	–	–	−8,629, *p* < 0.001
*Ambiguous*	4	6.518, *p* < 0.001	–	–	−3.758, *p* < 0.01	4.205, *p* < 0.01	−7.272, *p* < 0.001
	5	6.571, *p* < 0.001	–	3.547, *p* < 0.05	−3.698, *p* < 0.05	4.803, *p* < 0.001	−3.893, *p* < 0.05
	6	4.471, *p* < 0.001	–	–	–	5.739, *p* < 0.001	–
	7	–	–	–	–	9.490, *p* < 0.001	–
*/ø/−like*	8	–	–	–	–	11.877, *p* < 0.001	–
	9	–	–	–	–	10.391, *p* < 0.001	–
	10	–	–	–	–	13.210, *p* < 0.001	–

*Data are presented in terms of experimental groups (cases where no significant difference is found are denoted by the symbol “–”)*.

While both child groups were affected by both AV conditions when they perceived ambiguous auditory stimuli (stimuli 4–7), Table 3 shows that, compared to C1, C2’s responses were more influenced by the visual inputs. Furthermore, for C2, visual influence tended to become more pronounced as a function of the distance from the auditory stimulus. Interestingly, this phenomenon also extends to the endpoint stimuli.

*Post-hoc* tests revealed that C2’s responses were also affected when incongruent visual inputs were presented with close-to-endpoint auditory stimuli (highlighted in dark gray in Table 3). As Figure 3 (right-hand panel) reveals, when /e/-like auditory stimuli (1–3) were presented with the /ø/ visual articulation (dark gray line), children in C2 identified most of the targets as /ø/. Likewise, when /ø/−like auditory stimuli (8–10) were perceived simultaneously with the visual articulation of the vowel /e/ (light gray line), C2 children generally disregarded auditory cues and based their decisions primarily on the visual input. Thus, in contexts where acoustic and visual information were incongruent, the contribution of visual articulatory gestures played a leading role for children in C2 (see Figure 4) such that no phonological change was observed over the course of the auditory continuum, regardless of the clarity of the auditory stimulus.

To further explore the dissimilarities between the two groups of children, additional LMEMs were run and confirmed that behavioral differences between the two groups were not due to sex [*χ*^2^(1) = 0.039, *p* > 0.5] or age [*χ*^2^(1) = 0.002, *p* > 0.5]. Moreover, no differences between C1 and C2 were found in the AO condition.

Finally, independent *t*-tests were performed on the mean standard error of every stimulus, in each of the three experimental conditions. None of the conditions led to significant differences in variability between C1 and C2 responses [AO: *t*(12.411) = −0.321, *p* < 0.05; AV/e/: *t*(18) = −0.762, *p* < 0.05; AV/ø/: *t*(10.854) = 0.667, *p* < 0.05]. Moreover, when comparing variability of responses to endpoint stimuli, no significant differences were detected between the two groups of children, regardless of whether the auditory and visual information were compatible [*t*(21) = −0.956, *p* > 0.05] or not [*t*(21) = −1.264, *p* < 0.05]. Greater variability among C2 children could have been an indication of an attentional bias.

While no variability differences were found between the two child groups, the data show that child participants were generally more variable than adults in both congruent [Adults vs. C1: *t*(11.194) = −42.990, *p* < 0.05, Adults vs. C2: *t*(12.406) = −4.423, *p* < 0.001] and incongruent AV conditions [Adults vs. C1: *t*(36) = −39.742, *p* < 0.01, Adults vs. C2: *t*(37) = −5.242, *p* < 0.001].

## Discussion

The goal of this study was to investigate whether the audiovisual perception of speech differs between preschool-aged children and adults. We chose to investigate the rounding feature, which is the most visually salient, and focused on the perception of the French vowel pair /e/ and /ø/ in 27 adults and 23 preschool-aged children. Our results suggest that the visual influence on auditory perception may occur early in development, but its effect on phonological categorization differs in children and adults. This mechanism likely requires mature sensory systems and sensorimotor representations.

### Audiovisual Interaction in Perception

It has been shown that perception is usually facilitated by congruent auditory and visual presentation. Indeed, according to the intersensory redundancy hypothesis, concordance of multiple signals guides attention, reduces RT, and, ultimately, disambiguates perceptual processing (Bahrick and Lickliter, 2000, 2012). Although, no major impact of visual information was found in our data when auditory and visual information were compatible (probably due to a ceiling effect), Figure 3 suggests that endpoint stimuli (stable ones) were classified either similarly or better with congruent visual cues.

Bimodal presentation also facilitates perception when one of the sensory sources is damaged or unstable (Barutchu et al., 2010; Ross et al., 2011). In that case, additional cues help recover the weaker signal. In our study, a similar outcome was observed when optimal visual inputs were perceived simultaneously with ambiguous auditory stimuli. Indeed, although the three experimental groups were not affected to the same extent by the different visual inputs, all participants’ categorizations of ambiguous auditory percepts were influenced by the visible articulatory movements. This could mean that the auditory and visual sensory modalities interact in the perception of speech targets in children and adults, either when both sensory sources are compatible or when one of them (in our case, auditory) is imprecise.

Yet a disparity in our results is observed when participants perceived auditory and visual information sources that were incompatible. As shown in Figure 3, when adults (left-hand panel) and children in C1 (middle panel) perceived /e/-like stimuli (1–3) combined with the visual input for /ø/ (dark gray lines), the overall percentage of perceived /e/ barely decreased. However, a massive change in overall categorization was observed in C2 (right-hand panel). The same effect was found when /ø/-like auditory stimuli (8–10) were presented with visual articulatory movements for /e/ (light gray lines): trivial changes were observed in adults and C1 while a substantial increase in the perception of /e/ was found in C2.

As Burr and Gori (2012) suggested, the first 8 years of life are critical for brain plasticity. During that period, experiences and comparisons are used to calibrate our senses in order to benefit from them. According to cross-modal calibration theory, before optimal integration abilities are achieved, the most robust sense prevails over the others (Burr and Gori, 2012). In neurotypical individuals, once brain maturation has occurred, cross-calibration gives way to multisensory facilitation. At that point, when confronted with hard-to-define inputs (ambiguous, noisy, and poor quality), the sturdiest sensory modality has more weight in the perceptual process (Burr and Gori, 2012). In our study, the results of bimodal perception of ambiguous auditory targets in adults and C1 children, for whom overall categorization was influenced by visible speech movements, are in line with this hypothesis. Although the classification patterns of the adult and C1 groups are similar, the fact that the responses of children in C1 were more variable than the adults’ may indicate that their cross-modal calibration is still being refined.

Consequently, the children from the C2 group, who based their decisions on visual cues when unrelated bimodal information was presented, may have done so because they used vision as the calibrator in incongruent speech processing. As Burr and Gori (2012) mentioned, the calibrator is not necessarily the most precise sense but the most robust one. Since the vowel targets used in this study contrasted in terms of rounding, and this feature is the most visually salient one in French (Robert-Ribes et al., 1998), it is possible that this particular group of children, who had not yet achieved adult-like MSI, chose to rely on this contextually stronger sensory signal. The results of Kushnerenko et al.’s (2008) study could also be interpreted in that way. They found that 5 month olds processed “auditory /ga/ visual /ba/” as a mismatched signal but not “auditory /ba/ visual /ga/.” Because the consonant [b] is produced in a more visually salient way than [g], it could be that infants were more influenced by the visual information when it contained prominent cues.

Of course, multisensory perceptual tasks generally require increased attention. Contrary to the facilitation effect resulting from congruent signals, the increased difficulty and need for sustained attention is often cited as the reasons for children’s poorer scores in atypical multisensory conditions, such as reduced SNR (Spence and McDonald, 2004; Alsius et al., 2005; Barutchu et al., 2009). If greater variability had been identified in C2’s responses than in C1’s in the incongruent AV presentation (/e/-like auditory + /ø/ visual or /ø/-like auditory + /e/ visual), divergent categorizations between the two groups of children could be explained (at least partially) by an attention bias. However, our comparison of the variability of responses indicated that both groups of children showed similar patterns of variance, in both congruent and incongruent conditions.

Hence, taking into account the relative weight of visual and auditory information for phonemic identification, the cross-calibration hypothesis seems to offer an interesting explanation of why some of the children based their decisions on the visual input, even though audition is considered to be the dominant type of sensory information in speech perception mechanisms. Importantly, the fact that no adult acted similarly to the C2 individuals and that no differences in variability were found between the two groups of children lead us to believe that the differences in categorization are due to a developmental variable, rather than an individual one.

### Sensorimotor Maturation

The group difference in terms of audiovisual perception could, however, also reflect the maturation of sensorimotor representations of speech (see Caudrelier et al., 2019). Indeed, children with more mature articulatory speech production patterns for certain articulators would attribute more weight to these articulators at the perceptual level.

The link between motor experience, its sensory consequences, and children’s still developing feedforward models, while less often applied to the domain of speech development, is well established in the field of motor control, particularly with regard to arm movements. Children’s gestures are known to be slower, less precise, and more inconsistent than adults’, due to their lack of sensory experience (Jansen-Osmann et al., 2002; Lambert and Bard, 2005). A recent study of speech motor control maturity in preschool-aged francophone children also acknowledges the role of underspecified sensorimotor maps in explaining inaccurate and unstable predictions of speech motor commands (Barbier et al., 2020). The authors conclude that the onset of maturation and sensorimotor development occurs at around 4 years old.

In our experiment, this could mean that unlike children from the C1 group, children from the C2 group still have incomplete sensorimotor maps. Thus, they might have well-defined articulatory patterns for the lips, but not yet for, say, the tongue. Consequently, they would assign more weight to perceptual information concerning aperture, lip rounding, and lip stretching which is transmitted more prominently through the visual channel. This is in line with the hypothesis that immature sensorimotor representations can interfere with multisensory processing in speech. We believe that this hypothesis is not incompatible with the cross-modal calibration hypothesis and that it may even underlie it.

### Limitations of the Study

A potential variable that was not taken into account in this study is between-subject variability in terms of lip-reading skills. Given that this competence evolves and is refined throughout childhood, a precise measurement of lip-reading skills could have provided a more detailed account of the children’s perceptual responses. Furthermore, although we chose to use prototypical visual presentations of the /e/ and /ø/ vowels in combination with prototypical and ambiguous auditory stimuli, the use of ambiguous visual information (intermediate degrees of rounding between /e/ and /ø/) in combination with the auditory stimuli could help refine our analyses of children’s perceptual responses. Indeed, in cases of visually ambiguous stimuli, reliance on auditory cues might be enhanced in children as it is in adults. Follow-up studies are currently underway to further investigate these issues.

## Conclusion

This study investigated whether the visual modality influences the auditory perception of the French rounded vowels /e/ and /ø/ in preschool-aged children in the same way as in adults. Our results suggest two distinct patterns of response for children. In the first group, visual influence on auditory perception is similar to (or greater than) that of adults, while in the second group, responses are dominated by the visual content of the stimuli. Thus, substantial individual differences in audiovisual perception still exist at that stage. We suggest that auditory and visual speech perception skills are still developing around that age and that multisensory processing took place only for children whose sensory systems and sensorimotor representations were mature.

## Data Availability Statement

The raw data supporting the conclusions of this article will be made available by the authors, without undue reservation.

## Ethics Statement

The studies involving human participants were reviewed and approved by the Université du Québec à Montréal’s Institutional Review Board (no. 2015-05-4.2). Written informed consent to participate in this study was provided by the participants’ legal guardian/next of kin.

## Author Contributions

PT-F, LA, and LM contributed to the conception and design of the study. PT-F collected the data, organized the database, performed the statistical analysis (all under LM’s guidance), and wrote the first draft of the manuscript. PT-F and LM were invested in subsequent drafts of the manuscript and contributed to manuscript revision, read, and approved the submitted version. All authors contributed to the article and approved the submitted version.

## Funding

This work was funded by the Social Sciences and Humanities Research Council of Canada (Canadian Graduate Scholarships—a Doctoral program and an Insight grant) and the Natural Sciences and Engineering Research Council of Canada (a Discovery grant).

## Conflict of Interest

The authors declare that the research was conducted in the absence of any commercial or financial relationships that could be construed as a potential conflict of interest.

## Publisher’s Note

All claims expressed in this article are solely those of the authors and do not necessarily represent those of their affiliated organizations, or those of the publisher, the editors and the reviewers. Any product that may be evaluated in this article, or claim that may be made by its manufacturer, is not guaranteed or endorsed by the publisher.
